# Cough, with Vomiting

**Published:** 1882-05

**Authors:** E. B. Nash

**Affiliations:** Cortland, N. Y.


### COUGH WITH VOMITING.

E. B. NASH, M. D., CORTLAND, N. Y.

*Anacardium.*—Cough after eating, with vomiting of food.

*Bryon.*—Cough after eating or drinking with vomiting of ingesta, worse on coming into a warm room.

*Carbo veg.*—Cough and vomiting after other symptoms of whooping-cough are gone, especially after full meal.

*Daph. ind.*—Cough with vomiting and yellowish frothy expectoration, mixed sometimes with streaks of blood; the cough fatigues and hinders sleep.

*Digital.*—Cough after a meal, with vomiting of food, especially after cold fluids; pains in the arms and shoulders.

*Drosera.*—Cough with vomiting of food first, and later, at the end of the attack, of mucus.

*Ferrum.*—Spasmodic cough, ceasing immediately after a meal, or else commencing after a meal, with vomiting of food.

*Ipecac.*—Continuous cough, with sweat on the forehead, shocks in head, retching and vomiting (*Pertussis*).

*Kali carb.*—Spasmodic cough, with gagging and vomiting of ingesta and sour phlegm (3 A. M.)

*Mezereum.*—Cough when drinking or eating anything hot; must cough until food is vomited.

*Phos. ac.*—Cough, with vomiting of food, and headache, or involuntary urination.

*Rhus tox.*—Cough with vomiting of the ingesta, especially in the evening, and when lying on the back.

*Sabadilla.*—Cough dry, with perspiration and water in the eyes; stitches in vertex, vomiting and pain in the stomach.

*Sepia.*—Paroxysms of spasmodic cough (resembling whooping-cough), ending in gagging or vomiting.

*Tart. em.*—Cough if children get angry; also after eating, vomits food and mucus; perspiration on hands and forehead.

